# Challenges and opportunities for precision medicine in neurodevelopmental disorders

**DOI:** 10.1016/j.addr.2022.114564

**Published:** 2022-09-29

**Authors:** George T. Chen, Daniel H. Geschwind

**Affiliations:** a Department of Neurology, David Geffen School of Medicine, UCLA, United States; b Center for Autism Research and Treatment, Semel Institute, David Geffen School of Medicine, UCLA, United States; c Department of Psychiatry and Biobehavioral Sciences, Semel Institute, David Geffen School of Medicine, UCLA, United States; d Department of Human Genetics, David Geffen School of Medicine, UCLA, United States; e Institute of Precision Health, UCLA, United States

**Keywords:** Neurodevelopmental disorders, Precision medicine, Autism spectrum disorders, Schizophrenia, Neurodevelopment, Gene therapy

## Abstract

Neurodevelopmental Disorders (NDDs) encompass a broad spectrum of disorders, linked because of their origins in brain developmental processes, including diverse conditions across the age span, including autism spectrum disorders (ASD) and schizophrenia (SCZ). Clinical treatment of these disorders has traditionally focused on symptom management, as the severity of developmental disruption varies widely and the precise molecular mechanisms, timing, and progression of these disorders is usually not known. Several hundred genes have been identified as major risk factors for ASD and SCZ, which creates new potential therapeutic avenues, and there is strong evidence that these genes converge upon key molecular pathways, pointing to opportunities for precision medicine. In this review, we focus on forms of ASD and SCZ with known genetic etiologies and discuss advances in research technologies that enable a more systemic understanding of disease progression. We highlight recent advances in targeted clinical treatment and discuss ongoing preclinical efforts as well as new initiatives aimed at developing scalable platforms for NDD precision medicine.

## 1. Introduction

**Fig. 1.**
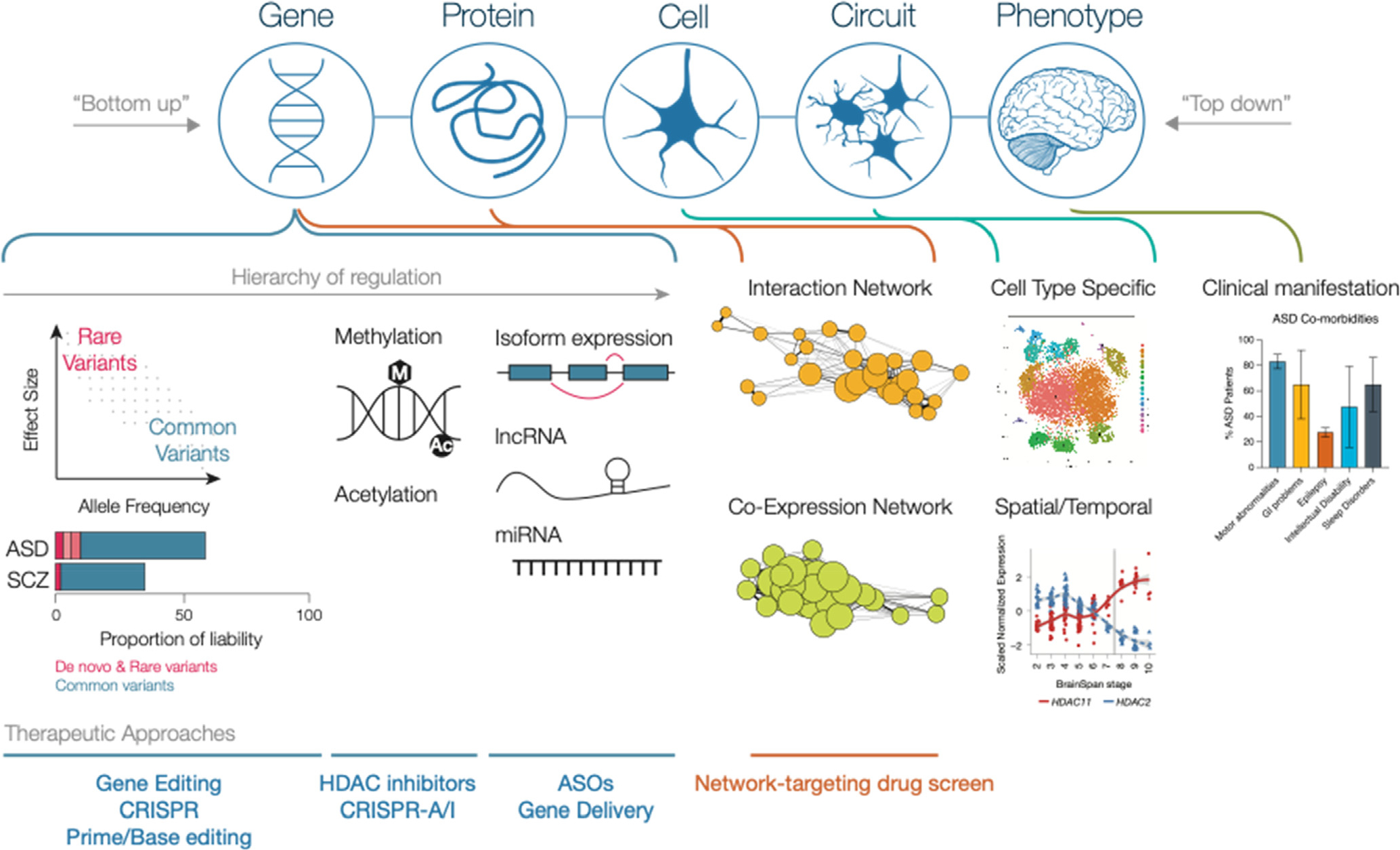
Organizational framework for NDD pathogenesis and levels of analysis. At the gene level, multiple layers of gene expression regulation have been shown to be relevant in NDD progression. Common variants underlie most of the disease risk but individually have low effect; rare mutations elicit more severe effects [44,22]. Additionally, methylation and acetylation have both been shown to regulate expression of ASD related genes. At the transcript level, additional forms of regulation, including alternative splicing, lncRNA, and miRNA modulate gene expression. The expression and interaction of genes and proteins can be explored using interaction networks (eg. PPI) and co-expression networks (eg. WGCNA) to identify biological processes and key hub genes that may prove to be attractive clinical targets. At a cell and circuit level, single cell analysis of human fetal brain [72] illustrate distinct cell types present in the developing brain. Furthermore, analyses conducted across time and between different brain regions reveal spatial and temporal differences in gene expression – for instance, a switch between expression of *HDAC11* and *HDAC2* in the developing prefrontal cortex as shown in the Brainspan transcriptomic data; the grey line denotes the switch from prenatal to postnatal periods [29]. In the clinic, ASD patients present with a number of co-morbidities; these may be useful as biomarkers for drug development as those with a defined phenotype can be used to screen for drug efficacy [4,8]. Therapeutic approaches can be organized within the framework by target; while gene editing approaches target individual genes or variants, network-based drug screens can link gene co-expression networks to cellular processes and circuits.

Neurodevelopmental Disorders (NDD) represent a diverse compendium of disorders, encompassing many conditions which impair cognitive or motor function development [95]. With a few exceptions, such as SCZ which has a later onset, these disorders typically display an early onset during infancy or childhood, involve the impairment or delay in maturation of the central nervous system, and exhibit a steady course of disease, rather than remission/relapse cycles present in other mental disorders. Importantly, NDDs often co-occur, highlighting the possibility of shared or overlapping biological processes in affected individuals. ASD is a prototypical NDD, as it has an onset in early development and etiologic studies causally implicate impaired early neurodevelopmental processes that impact an individual across their lifetime [56]. SCZ, although phenotypically could be classified under disruptive behavior disorders, is also classified as an NDD because its mechanisms are increasingly recognized as neurodevelopmental; additional support for this classification comes from the significant genetic overlap with ASD [21,64]. In fact, shared genetic risk factors for multiple major neuropsychiatric conditions, including those with adult onset such as Major Depressive Disorder (MDD), Alcohol Abuse Disorder, and childhood onset disorders such as Attention Deficit Hyperactivity Disorder (ADHD), also impact neurodevelopmental pathways [88,49], including neurogenesis and synaptic transmission. Therefore, at some level, neurodevelopmental processes contribute broad risk across neuropsychiatric disorders (see Fig. 1).

Here we focus on two NDDs, ASD and SCZ, which have shared genetic risk, environmental risk factors, and a high rate of co-occurrence [111]. The precise number of individuals affected with neurodevelopmental disorders is difficult to predict; the most recent estimates in the United States report 1 in 54 children are affected by autism spectrum disorders [59] and 0.3–0.6 % of individuals globally by schizophrenia [28,26], though these numbers are not exclusive of one another. The underlying challenge in both identifying and treating these disorders is the heterogeneity that exists at multiple levels: in the presentation of the disorder, severity of symptoms, comorbidities present, and expression of symptoms over time. Furthermore, the clinical diagnosis of NDDs is defined by symptom inventories, which provide little information as to the affected genes or molecular mechanism. To this end, the National Institute of Mental Health (NIMH) has made efforts to establish a new framework to define psychiatric disorders based on their neurobiology [38]. However, connecting this framework to specific genes has been challenging, particularly in NDDs which are largely polygenic and in which gene-phenotype linkages are weak [85]. A recent review [63] of commercial gene panels marketed for ASD detection found that while these panels generally covered known high confidence ASD risk genes [87,81], there still is much to be learned. The diagnostic yield of these panels on the SPARK cohort of ASD individuals [17] was between 0.22 and 10 %. While 10 % detection is quite remarkable for a single test, and comparable to results from whole exome sequencing, most patients would have been missed. Thus, there has been limited advancement in developing targeted diagnostic tools and therapies, despite hundreds of risk genes having been identified over the last decade [27,84] and promising preclinical results in monogenic and syndromic forms of NDDs. Unfortunately, the most recent well-powered clinical trials targeting individuals with Fragile X syndrome have yielded disappointing results [16,39], highlighting that even for rare monogenic or syndromic forms of NDDs, there remains additional complications to consider. To date, clinical treatment continues to be limited to the management of symptoms [42,5].

In this review, we will discuss key processes and milestones that need to be reached for precision medicine to be successful in the treatment of NDDs – 1) large-scale genomic studies to identify useful biomarkers, 2) efficient means of patient stratification tied to causal mechanisms, and 3) an integrative approach to translation [18]. We discuss progress in each of these over the past decade, highlight approaches to transition to the clinic, and discuss the benefits and drawbacks of various gene therapy platforms.

## 2. Genetic and genomic studies reveal convergent mechanisms of disease

The underlying challenge in identifying risk genes for NDDs is that most do not arise from single mutations; rather, common genetic variation underlies most of the risk at the population level (ASD: [44,24], SCZ: [50]). This polygenic basis underlies the vast heterogeneity observed in patients - in clinical phenotypes, chronology of development, and response to treatment [55]. Such multi-level variation suggests the need to treat each disease as a separate entity or to stratify cases based upon defined criteria, eg. subtypes. The most obvious initial subtype in ASD would be patients with *de novo*, large effect mutations, which comprise approximately 10–20 % of cases [44] and are likely not as significantly influenced by polygenic risk. However, the majority of cases appear to be driven by polygenic risk and lower effect size rare variants [104,81,89,27]. To subset these cases requires large-scale genomic data to achieve sufficient statistical power given the extreme heterogeneity between patients, and a network-based, bottom up approach [64] to identify affected mechanisms and pathways in lieu of individual driver genes.

Significant progress has been made using network-based approaches over the last decade in understanding convergent and unique mechanisms between NDDs, spatiotemporal differences during neurodevelopment, and how dysregulation of brain developmental processes lead to common clinical phenotypes. Importantly, these studies have focused on different modes and levels of neurodevelopmental regulation (Table 1). In Voineagu et al. [98], postmortem brain samples from three different regions were sequenced in ASD cases and controls. Supervised hierarchical clustering showed a distinct clustering of ASD cases compared to controls and was independent of clinical phenotypes (age, sex, seizures, medication). Using Weighted Gene Co-expression Network Analysis (WGCNA) [46], the authors identified two gene co-expression modules that were associated with ASD – one containing significant enrichment for neuronal markers was downregulated in ASD cases, and another module enriched for astrocyte markers and activated microglia was upregulated in ASD cases compared to controls. WGCNA creates these modules by grouping genes by co-expression, such that genes that are co-regulated cluster together; importantly this co-regulation is related to shared biological processes by the genes in that module [15,45,47]. Using GWAS datasets for ASD cases, the authors demonstrated the neuronal development module to be significantly enriched in ASD cases, while the immune activation module was not enriched, indicating that this upregulation was not through a genetic component.

Parikshak et al. [65] took a different approach, starting with causal risk genes (bottom up from a genetic standpoint) rather than pathological tissue (top-down or phenotypic) from patients who had a diagnosis of ASD, performing WGCNA on neurotypical bulk RNAseq data from fetal brain. Of the co-expression modules identified, three modules were found to be significantly overrepresented with known ASD risk genes identified in previous genetic studies collated by the Simons Foundation Autism Research Initiative database [7]. These modules were enriched in gene ontology terms related to early neuronal/synaptic development and share closely aligned, but distinct developmental trajectories. The ASD-upregulated module identified by Voineagu [98] was also strongly enriched in these neuronal modules. Notably, high confidence genes implicated in monogenic forms of intellectual disability (ID) did not show a specific enrichment linked to any of the modules, highlighting the specificity of these modules to ASD. ASD genes were also shown to be most consistently associated with upper layer glutamatergic neurons (Layer 2/3 in primates), suggesting that these cells are most affected by ASD mutations. Using an alternate approach on the same dataset to create gene co-expression networks, Willsey et al. [106] started with nine high confidence ASD genes as “seeds” to build a spatiotemporal co-expression network. These networks identified enrichment in ASD risk gene expression in the developing prefrontal cortex and specifically identified dysregulation of deep layer projection neurons from data in mouse (Layer 5/6) in the pathogenesis of ASD, though recent PPI analysis [70], as we discuss below, supports a stronger ASD signal in upper layer neurons, emphasizing the importance of multi-omic approaches in elucidating specifically affected cell populations.

MicroRNA (miRNA) expression profiling of postmortem ASD brains [108] also identified co-expression modules significantly correlated with ASD, but not ID. Consistent with observations in mRNA, the expression profiles were very similar between the prefrontal and temporal cortex in ASD samples. This study highlighted the fact that ASD risk genes are highly dosage sensitive, as the additional gene expression regulation conferred by aberrant miRNA expression was able to perturb neuronal development. Furthermore, a histone acetylome-wide association study of postmortem ASD brains and matched controls revealed a common acetylome mutation signature in the prefrontal and temporal cortex [91] of idiopathic and syndromic samples. This analysis identified histone acetylation quantitative trait loci (haQTLs) that mark region-specific regulatory variation and revealed specific haQTLs strongly linked to ASD and SCZ variants.

Multi-omic characterization of ASD postmortem brains collectively show dysregulation of genes involved in neuronal activity and synaptic transmission and an upregulation of genes involved in astrocytes and inflammation. However, this shared pattern is present only in approximately two-thirds of the samples. To better understand how epigenetic changes regulate expression of their cognate genes and to identify ASD subtypes that clarify this heterogeneity, Ramaswami et al. used Similarity Network Fusion (SNF) [101] to integrate mRNA expression, miRNA expression, DNA methylation, and histone acetylation datasets [76]. Using this approach, two ASD subtypes were defined based upon convergence of transcriptomic and epigenomic signatures. Samples that showed convergence collectively pointed to a model in which ASD risk variants perturb regulatory elements of genes identified in Voineagu module M16 [98], leading to a downregulation of synaptic signaling which in turn leads to transcriptional upregulation of astrocyte and microglial immune processes via a decrease in DNA methylation and miRNA expression. By contrast, samples that excluded from this subtype collectively showed no differentially expressed genes, co-expression modules, differentially expressed miRNA, or differentially methylated promoters compared to neurotypical controls, suggesting that these samples differ in their molecular dysregulation and additional samples are needed to further characterize additional ASD subtypes.

While these analyses have focused on gene expression regulation, dysregulation at the protein interaction level in NDDs have been less studied. Using a complementary approach, Pintacuda et al. [71] and Hsu et al. [36] used induced neuron cultures and immunoprecipitation to capture interactors of high confidence ASD and SCZ risk genes, respectively, to generate an integrated protein-protein interaction map for each disorder. The resulting maps showed high connectivity between the genes of interest, providing further evidence for convergence in affected gene processes despite disparate initiating variants. The ASD map was enriched in genes observed in layer 2/3 cortical neurons, while the SCZ map was specifically downregulated in layer 5/6 cortical neurons. These maps identified essential proteins and complexes that may be prioritized for targeted treatment, including a subnetwork centered around *HCN1* that is enriched for common variant risk in SCZ.

Given the shared disruption of neurodevelopmental processes among NDDs, it is unsurprising that there is considerable overlap between disorders in transcriptional dysregulation, the degree of which is related to the extent of polygenic overlap between disorders. In a study of transcriptomes from over 1600 postmortem brain samples from ASD, bipolar disorder (BD), SCZ, and neurotypical patients [21], differentially expressed genes between each disorder and matched controls showed significant cross-disorder overlap, with the largest changes in expression in ASD compared to SCZ or BD. Differential transcript expression showed more of a disease-specific signal, suggesting that alternative splicing or alternate transcript usage explains a substantial portion of disease specificity. WGCNA was performed to identify gene-level and isoform-level modules, most of which showed significant association to at least one disorder, and nearly half showed strong cell type enrichment. Several of the modules were shared across all three disorders, representing an increase in inflammatory signaling and excitatory neuron signaling and an impairment of blood-brain barrier regulation. One example of disease-specific neural immune dysregulation included microglia, which were only observed to be up-regulated significantly in ASD. These findings were similar to an earlier study combining microarray data from additional neuropsychiatric disorders [23], where SCZ and BD showed the greatest similarities in transcriptome, followed by ASD with either SCZ or BD. These results help establish a framework for understanding the variation in effect upon mechanisms common across disorders.

## 3. Using transcriptomic patterns or gene networks for drug discovery

We [92,77,12] and others [86] have leveraged transcriptomic patterns identified in disease models to take a gene-group or network-based approach in neural repair and neurodegenerative disease studies to identify drugs that reverse specific neural phenotypes. Using network approaches allows for the identification of highly-connected, or hub, genes central to a phenotype whose modulation is most likely to cascade in effect across a network. These conditions have the advantage over ASD or SCZ in that once we identify drugs, we know which cellular and molecular phenotypes to screen for correcting (e.g., axon outgrowth or neuronal survival). Thus, we performed proof-of-principle studies in neurodegeneration and neural repair to test the predictive power of this approach.

Combining data from multiple mouse models of neurodegeneration, Swarup et al. [92] used WGCNA to identify evolutionarily-conserved, disease-relevant gene co-expression modules. Network analysis of miRNA expression identified miR-203 as a hub gene for a disease-linked module. *In vivo* overexpression of miR-203 showed it regulates neuronal genes and increases apoptosis. The observed module expression changes were screened against a public database of cell line responses to drugs to identify HDAC inhibitors as likely to reverse module changes. *In vitro* testing of two of the predicted HDAC inhibitor compounds ameliorated gene expression changes in the predicted directions and more importantly, demonstrated decreased neuronal death. In another neurodegeneration study, Rexach et al. focused on microglia purified from mouse and human samples of tauopathies combined with bulk tissue data [77] and identified modules with distinct temporal expression trajectories marking microglia transitions across disease progression. Two distinct and opposing modules were found to be upregulated early in disease; one activating immune response and the other suppressing immune response. Compounds predicted to drive expression of these modules were tested *in vitro* and demonstrated the functional link between these two modules and a refined understanding of microglia signaling early in neurodegeneration. These two studies demonstrate the power of network approaches to identify collective effects of differentially expressed genes, observe temporal changes in cell processes, and use these observations to inform putative therapeutic targets.

We also emphasize that in addition to the challenge of having biologically relevant phenotypes to correct in NDDs, postmortem population-scale studies represent molecular phenotypes at fixed states. Complemented with brain development studies and focused studies on rare monogenic and syndromic forms of NDDs, these studies have collectively identified common and unique mechanisms across NDDs and specific cell types and developmental periods that are affected (Table 1). However, what remains unknown are the degree to which the effects of these mechanisms can be targeted for treatment, whether such treatments must occur in fixed periods of developmental time, and whether treatments can be broadly applied across the spectrum of patient phenotypes. The studies that have attempted to functionally correct NDD symptoms have focused on monogenic and syndromic variants which exhibit the most severe phenotypes. Though the postmortem gene co-expression networks suggest convergence in dysregulated processes between rare variants and polygenic common variants compared to neurotypical controls, the degree to which findings in monogenic forms of NDDs inform our understanding of polygenic forms remains an open question. Furthermore, genetic and phenotypic stratification of patients is necessary to identify clinically relevant populations, as heterogeneity is widely evident even in monogenic NDDs.

## 4. Proteomic analyses point to shared systemic pathology between ASD and SCZ

Complementary to transcriptomic analyses, proteomics hold the potential for use in screening and diagnosis; particularly in delineating between different NDD subtypes. There is substantial evidence that developmental pathways disrupted in NDDs also contribute to systemic changes in development [4]. Such dysregulation has been identified in proteomic studies of ASD [2] and SCZ [82] patient sera, which both showed an increase in inflammation, particularly in acute phase response, and lipid metabolism compared to neurotypical individuals. These findings have already been observed at the transcriptome level, though these proteomic analyses may point to additional levels of expression regulation. However, current proteomic studies suffer from significantly smaller patient populations compared to transcriptomic datasets; until more replication studies can be conducted, their utility as screening biomarkers is limited.

## 5. Stratification of patients requires multimodal processes

An underlying challenge in performing clinical trials for the treatment of NDDs is the lack of objective, reproducible treatment endpoints. Given the wide heterogeneity in phenotypes for each disorder, a reasonable approach would be to define endpoints that specifically capture non-psychiatric symptoms that affect quality of life but may be more easily quantifiable compared to more complex cognitive, social, or behavioral measures. These endpoints should be informed by the mechanisms elucidated from genetic and neurobiological studies and ideally paired with clinical genotyping. Additionally, the population level network analyses we previously discussed suggest that genotypic information should be used to subset patients by shared disruption of a characterized network pathway or module, as the polygenic nature of NDDs for most individuals makes the utility of sub-setting on a specific variant unreliable. It is important to consider, based upon the wide heterogeneity of NDD presentations, that both endpoints and subset criteria need to balance between being broad enough to have a large enough study group and specific enough that the observed effect will have enough statistical power.

Current diagnostic modalities for NDDs still largely rely upon behavioral phenotypes and clinical observation, with genotypic information typically not factored into diagnosis. Such observations have limited utility in stratifying patients in the absence of complementary biomarker data; likewise, the ability to predict future outcomes requires markers with temporal specificity. Unfortunately to date, no biomarkers have been identified that are either prognostic or predictive of disease progression for the majority of ASD or SCZ cases.

However, there are notable exceptions for specific forms of ASD based upon genetic diagnosis including dup15q syndrome, a duplication of 15q11.2-q13.1 is associated with both ASD and intellectual disability [58,14] and Tuberous Sclerosis Complex, a noncancerous tumor-causing mutation in which approximately 50 % of affected individuals are diagnosed with ASD [10,100]. Given the complexities of NDDs, it is likely that combining biomarkers (eg. risk genes, EEG, MRI, metabolomics) will have a synergistic effect in stratification schema. Neuroimaging studies using whole cortex magnetoencephalography (MEG) have shown potential value in helping to link NDDs models with patient data [20] and in prognostic applications [79]. Gandal et al. [20] used whole cortex MEG to measure neurotypical and ASD children and compared the recordings to mice treated with valpronic acid, an insult-based model of ASD. The recordings revealed a correlation in deficits observed in ASD and treated mice compared to their associated controls. The same group proposed in Roberts et al. [79] to use the M50 auditory evoked response component detected by MEG as a biomarker for ASD. They were able to identify a subpopulation of patients with significantly exacerbated M50 latency delay with low levels of GABA, suggesting that treatment of this subpopulation with GABA agonists may be of clinical value compared to the ASD population as a whole. How these measurements can be combined with genetic data was illustrated in a recent Dup15q study which compared ASD and neurotypical individuals treated with GABA agonist midazolam using EEG [19]. Several 15q genes have been linked to linked to disease pathology, including *UBE3A* and GABA receptor subunits *GABRB3, GABRA5,* and *GABRG3*. The observed EEG signal in neurotypical patients treated with the GABA agonist was similar to Dup15q patients. This phenotype was independent of elevated *UBE3A* expression, suggesting that GABAergic activity plays an important role in Dup15.q cortical dynamics. The authors suggest this observation could be extended to other NDDs displaying GABAergic dysfunction. Similar multimodal approaches have been used in SCZ to propose potential biomarkers [90], identifying a multimodal component using MRI and MATRICS Consensus Cognitive Battery that correlates structural differences with functional phenotypes.

In identifying biomarkers with quantifiable treatment endpoints, one may also consider non-CNS disorders that may commonly arise with NDDs. Identifying convergent gene networks between disorders may reveal specific risk genes whose function in development ties its mutation to specific periods in developmental time, as was observed in a recent study of ASD and cognitive heart disease (CHD) [80]. Rosenthal et al. noted that while risk genes for ASD and CHD did not show significant overlap, a systems-level approach revealed overlap in gene networks that pointed to *SCN2A* expression in early development leading to abnormalities in the brain and the heart. As *SCN2A* is not expressed in the adult heart, this shared mutant phenotype indicates a more specific timing to the impact of *SCN2A* on development and suggests that potential therapeutic intervention may need to focus on this developmental period for greatest effect. One could expand this approach to other co-morbidities, allowing for the stratification of patients by co-morbidity as well as mechanism of function, which may guide treatment schedules and predict therapeutic responses [25,97].

Though they are beyond the scope of this review, it is important to acknowledge additional liabilities that may affect individual responses to targeted treatment, including genetic background and environmental risk factors. The genetic background represents the sum of common variants, which may act as a buffer that diminishes or increases the deleterious effects of rare variants. Environmental risk factors such as maternal infections, prenatal exposure to toxins, and immune system dysfunction during development are also associated with increased risk for NDDs [99,57,60]. High concordance rates of NDDs in dizygotic twins compared to singleton siblings suggests a specific role for the fetal environment in modulating risk in some specific cases [93]. These factors may add uncertainty to stratification schema, emphasizing the need to use multiple modalities in characterizing patients.

## 6. Modeling treatment using rare and syndromic forms of ASD

Because of the lack of quantifiable biomarkers as we have just discussed, modeling NDDs *in vitro* has focused on rare variant or syndromic forms of disease, as these forms more easily demonstrate a direct relationship between mechanism and phenotype. Recent advances in gene editing technology have enabled the accelerated development of stem cell lines not only to target identified risk genes to induce mutations, but also to recapitulate mutations in specific genes or chromosomal regions identified in patients [34]. Along with induced pluripotent stem cell (iPSC) lines derived from NDD patients [102], these cell lines have allowed for a more comprehensive understanding of NDD development and affected cell populations across developmental time through directed differentiation to neuronal cell types.

## 7. Advances in stem cell biology and *in vitro* systems

Over the last decade, advances in stem cell biology have led to the development of self-organizing, three-dimensional cell cultures of differentiating cell types to model organogenesis *in vitro*. These cultures, often referred to as organoids, accurately recapitulate the developmental stages and organizational structure of the representative organ. In the context of the brain, numerous organoid protocols have been developed, broadly split between brain region-specific organoids and undirected brain organoids [67,75,40]. While undirected protocols have been useful in broadly understanding neurogenesis, their inherent heterogeneity makes them less appealing as a model system for studying NDDs than region-specific protocols. Brain organoids have been shown to be able to be maintained in culture for upwards of one year, and recapitulate the transcriptome of the developing brain for both prenatal and postnatal time periods [29]. These organoids have been shown to be highly reproducible, with temporal changes in gene expression that mirror *in vivo* expression [110,96,74]. Importantly, recent studies have established organoids using iPSC lines haploinsufficient for a number of NDD risk genes, including *KMT5B, PTEN,* and *CHD8* [103,68]. Each haploinsufficient line established organoids that demonstrated abnormal phenotypes consistent with clinical observations of patients with the same haploinsufficiency, strongly supporting the use of organoids to model NDD development. The authors used single cell sequencing to identify changes in cell type proportion across time and compared to wildtype for each haploinsufficient line. Each mutation was found to accelerate early development of cortical neurons but enriched for different neuronal populations; *KMT5B* for excitatory deep layer neurons, *PTEN* for callosal neurons, and *CHD8* for inhibitory interneurons.

For highly penetrant polygenic disorders, such as ASD & SCZ-associated 22q11.2 deletion syndrome (22q11.2DS), patient-derived iPSC lines have been used to establish organoid cultures and have demonstrated the ability to recapitulate disease phenotypes *in vitro* [41]. This approach identified significantly differentially expressed genes across development compared to neurotypical organoids, defects in neuronal excitability by electrophysiology, and impaired calcium signaling by flow cytometry. Additionally, heterozygous loss of *DGCR8*, a gene within the 22q11.2 region, was found to be sufficient to phenocopy the functional defects of the 22q11.2DS neurons. Overexpression of *DGCR8* partially rescued calcium signaling in neurons, as did treatment with antipsychotics. This project demonstrated the potential for patient-derived organoids to drive target discovery as well as screen therapeutic approaches *in vitro*.

Beyond modeling specific regions of the brain, organoids can be used to study defects in regional or organ-to-organ cell processes. A critical process in the formation of the cerebral cortex is the migration of cells to establish neural circuits. This process can be modeled in the organoid system by separately differentiating organoids to cortical or striatal cell fates prior to allowing them to fuse, forming what is termed an assembloid. The assembloid displays the same migratory processes as *in vivo*, with extensive migration of cells from the striatal organoid to the cortical organoid [9,61]. Using assembloids generated from Timothy Syndrome patients, Birey et al. observed less efficient interneuron migration compared to neurotypical controls. Timothy Syndrome is caused by mutations in *CACNA1C* and leads to ASD and epilepsy among other developmental defects. Impaired migration was rescued using L-type calcium channel blockers. The modularity of the assembloid approach has been expanded to model cortical development with other brain regions and nervous systems [6]; paired with patient-derived iPSC lines they offer a means to study the effects of disorders on neurodevelopment at a systems level. When combined with advances in single cell sequencing [29], 3D immunostaining [61], and 3D electrophysiology [66], it is possible to trace neurodevelopmental processes and how they are disrupted in NDDs at a cellular resolution, enabling *in vitro* modeling that more easily translates to *in vivo* studies.

## 8. Advances in animal models of NDDs

Animal models have been used extensively to elucidate the behavioral phenotypes and morphologic changes related to mutations in specific genes or regions of interest for syndromic and highly penetrant forms of NDDs [44]. However, while key symptoms have been replicated in mouse models, none fully recapitulate the human disorder they are modeling [33]. In part, this may be a function of the differences between mice and humans in terms of genetic susceptibility to NDDs; rodent models show more flexibility to haploinsufficiency in gene expression compared to primates [43]. Additionally, cognitive and learning tests are difficult to interpret in mice, due to the multisensory inputs present in most experimental setups that lead to heterogeneous response. One novel solution is the use of virtual reality, which limits sensory input to visual cues and has been shown to be a highly reproducible measure of learning [3,83]. This assay has the additional advantage in that the same system can be used in human trials, thus providing a more direct correlation in outcome measures. The increased specificity of outcome measures will become increasingly important when studying idiopathic and polygenic forms of NDDs.

Despite their challenges, animal models can play useful roles in identifying effective therapeutic delivery mechanisms and timings. Recently, a study of Dravet Syndrome, which is most commonly caused by haploinsufficiency in *SCN1A*, found that rescue of *SCN1A* gene expression in both immature and mature interneurons was capable of rescuing excitability defects [13]. Using a *Scn1a*^*+/−*^ mice model, the group found that intracranial injection of P0 pups with the rescue construct partially attenuated seizures. In a similar vein, treatment of adult *Scn2a*^*KO/+*^ mice with a positive regulator of AMPA receptors rescued a hyperactivity phenotype [94]. In neurotypical development, both *SCN1A* and *SCN2A* reach their peak expression in early postnatal periods; these studies suggest that the therapeutic window for these genes and other risk genes involved in synapse formation and function may be relatively broad. However, risk genes affecting essential *in utero* development processes such as chromatin remodeling and transcription are likely to have a much smaller therapeutic window given dependent downstream processes.

## 9. Gene therapies for neurodevelopmental disorders

As we have discussed, the heterogeneity of phenotypes in NDDs presents critical challenges in identifying functional targets of therapeutic interest, and in defining a patient population both to identify those most likely to benefit from the targeted therapy, but also to establish a clinical trial protocol unhindered by the heterogeneity of the larger affected population. Assuming these challenges have been addressed, the timing, dosage, and delivery of any therapeutic is complicated by nonlinear gene expression levels across developmental time. This is particularly important, as a majority of the identified high confidence risk genes have been predicted to be haploinsufficient [37]; a lower-than-normal level of expression resulting in fitness-limiting defects while overexpression is cytotoxic [62]. Targeted treatments therefore cannot merely overexpress risk genes or gene products, rather, additional gene expression regulation is required. Furthermore, these genes tend to be involved in key developmental processes and vary in expression across developmental time [27]. Thus, the therapeutic window for a targeted treatment is likely dependent upon the target’s function in development. Despite these complications, gene therapies have already begun to find clinical utility.

The last decade has seen an exponential rise in the development of gene therapies, with the first approved gene therapies for human diseases now actively used in the clinic. These early therapies focus on monogenic diseases with a highly penetrant genetic cause, for which the mechanism of disease is well understood. Syndromic forms of NDDs, such as *SCN1A* and *SCN2A*, have shown highly promising pre-clinical data, as highlighted above. Broadly speaking, gene therapies can be categorized as either transient or permanent, in terms of whether or not the genome is edited [105]. In terms of transient therapies, antisense oligonucleotides (ASOs) have received the most attention, as several have been approved for clinical use, notably for treatment of Duchenne muscular dystrophy (DMD) and spinal muscular atrophy (SMA) [52,35]. ASOs are synthetic nucleic acid analogues that can alter gene expression through mediating exon skipping. In the case of DMD, several exons are dispensable, allowing ASOs to be used in patients with mutations amenable to exon skipping, namely those with frameshifting mutations within said dispensable exons. The ASO treatment restores the reading frame of the DMD transcript, producing a truncated but functional dystrophin isoform [1]. ASOs can alternatively be used to induce RNA degradation or RNA-induced silencing; given that more than 95 % of human genes are reported to undergo alternative splicing and transcriptional processes are commonly mutated in NDDs [73], ASOs are a highly promising therapeutic approach. Currently in clinical trials are ASOs targeting *Scn1a* (Dravet Syndrome, Clinical Trial #NCT04442295) [32] and *Ube3a* (Angelman Syndrome, Clinical Trial #NCT04259281). Additionally, there is promising data from mouse models of *Scn8a* [51] and *Scn2a* [53]; if adopted for clinical use, these ASOs would all require dosing across the patient’s lifespan.

Another transient gene editing approach, CRISPR/dCas9, has showed promising preclinical data but has not yet been tested in the clinic [78]. This approach uses a deactivated Cas9 protein linked to an effector domain to activate or inhibit gene expression (CRISPR-A/I). The ideal targets of this therapy are genes that are haploinsufficient or have gain of function mutations in which the coding sequence is too large to be readily delivered through adeno-associated viral vectors. As CRISPR/dCas9 uses the endogenous gene expression machinery by targeting transcription initiation or inhibition complexes to enhancer or promoter regions, it is subject to secondary regulation, which limits cell toxicity as the result of overexpression. In two studies targeting *Scn1a* promoters in haploinsufficient mice, both groups reported *Scn1a* expression to be restored to near normal levels and related phenotypes rescued [13,109]. Using different effector domains, other groups have demonstrated CRISPR-activation of C11orf46 to repair transcallosal dysconnectivity [69]. Other effector domains have also proven useful in the CRISPR-dCas system, including methylation editing to restore *FMR1* expression in Fragile X Syndrome-patient derived iPSCs [54]. As the endogenous gene expression machinery is used, CRISPR/dCas may avoid challenges with dosage faced by other therapeutics; the system could remain present in cells without risk of gene overexpression and activate or inhibit genes in relation to their normal expression across development.

Gene delivery is another transient approach to correcting loss of function mutations by delivering complementary DNA (cDNA) for a particular gene. It has already been tested in monogenic forms of ASD including Fragile-X Syndrome, Angelman’s Syndrome, and Rett Syndrome both *in vitro* and *in vivo* [105]. In each of these studies, cDNA was packaged into an adeno-associated virus (AAV), which infects cells and delivers the cDNA to the nucleus to be transcribed. The introduced DNA is typically lost over subsequent cell divisions but has been shown to remain present in mature neurons, suggesting that this approach could be a long-lasting therapeutic solution.

In contrast to the ASO, CRISPR/dCas9, and gene delivery approaches we have discussed, gene editing approaches directly modify the genome. There are a limited number of examples for clinical use of gene editing in humans to date, though the approach has been successfully used for thousands of targets in preclinical models [11]. While the challenges of implementation are greater than ASOs – in particular, the need to guard against off-target effects and the delivery of relatively large nucleic acid payloads reliably to cell types of interest – the potential that gene editing can confer a long-term or potentially permanent therapeutic continues to drive interest in this approach. The most common methods used to perform gene editing are the CRISPR/Cas system, prime editing, and base editing. The latter two methods utilize a dead Cas linked to an engineered reverse transcriptase or DNA-modifying enzyme to induce a single strand cut and edit of single nucleotides through DNA mismatch repair. This method is preferable to CRISPR/Cas, which edits through non-homologous end joining or homology-directed repair, both of which have the potential to generate random, unwanted insertions or deletions. Proof-of-concept experiments using CRISPR/Cas on Rett Syndrome patient iPSC lines have shown success in correcting *MECP2* mutations [48] and in activating *UBE3A* in Angelman Syndrome [107].

Apart from ASOs, most gene therapies require viral vectors for delivery. Adeno-associated viruses (AAV) are particularly favored due to their lack of pathogenicity, ability to infect non-dividing cells, and lack of cytotoxic response from the host. AAVs can integrate DNA into the genome at specified regions, limiting the possibility of off-target events. Additionally, AAVs have multiple serotypes that confer tissue specificity, limiting the possibility of unintended consequences outside the nervous system. Recent research has improved upon serotype-linked tissue specificity to use enhancers of cell-type-specific markers to drive expression of fluorescent proteins, enabling stain-free imaging of neuronal cell subtypes [30]. Specifically, the authors synthesized three recombinant AAVs that labeled different projection neuron subtypes. This approach could be adapted to activate or inhibit gene expression in specific neuronal cell subpopulations affected by a specific NDD. Despite these useful characteristics, AAVs also have some drawbacks that need to be considered when developing therapies. AAVs have a relatively small payload limit (~4.8 kb), too small for classic CRISPR/Cas9 systems based on *S. pyogenes*, though alternative Cas enzymes such as *S. aureus* can avoid this limit. Over repeated cell divisions, AAVs diminish in concentration, thus may require repeated applications over an individual’s lifetime, though some evidence suggests recombinant AAVs can remain in mature neurons for several years [31] and recent evidence using AAVdelivered CRISPR/Cas9 demonstrated that AAV integration occurs at the double strand break site [107], potentially leading to a permanent edit of the genome.

## 10. Promise of precision medicine in neurodevelopmental disorders

The development of gene therapies has seen an exponential rise over the last decade, with the first approved gene therapies for human diseases now in clinical use. This rise has been enabled in part by advances in gene editing technologies as well as decreased genome sequencing costs, which has facilitated a fine-grained analysis of genetic mutations or variants leading to disease and the inheritance of such traits in the population as we have highlighted earlier. The modern gene editing toolkit provides a wide variety of potential therapeutic approaches that can be customized to the target gene of interest based upon its mutation – gene activation, inhibition, replacement, demethylation, prime editing, and base editing. The relative ease in developing precision medicine therapies compared to traditional pharmaceutical pipelines has drawn considerable interest from pharmaceutical companies as well as the National Institutes of Health. NIH has recently established the Bespoke Gene Therapy Consortium to focus on rare diseases and develop scalable experimental platforms and standards to accelerate future development. Specific to NDDs, the National Institute of Mental Health recently announced the Scalable and Systematic Neurobiology of Psychiatric and Neurodevelopmental Disorder Risk Genes (SSPsyGene) Consortium. The goals of this program are to perform functional analysis on ~100–200 high confidence risk genes and develop methods to standardize measures for future gene therapy targets.

As we have discussed, there are considerable challenges in developing precision therapies to target NDDs. Chief among these challenges is the polygenic nature of these disorders and the many potential causative mechanisms – inherited common variants, de novo mutations, copy number variation, and syndromic forms [44]. The heterogeneity in presentation, the severity of symptoms, the presence of co-morbidities, and the expression of symptoms over time collectively call for a precision-driven approach tailored to an individual’s disease presentation and genome. Thus far, progress has been made in using population-scale studies to identify gene co-expression modules shared between NDDs and unique to each disorder. The shared dysregulation in neurodevelopmental processes highlight shared pathways and modules of genes that may be suitable targets for therapeutic intervention. Advances in multimodal imaging approaches, paired with genomic data, offer the opportunity to stratify patients more selectively and establish objective measures for proving therapeutic efficacy, as opposed to current stratification based on behavioral phenotypes. The ability to more effectively cluster patients will allow for more targeted treatment that is more likely to succeed in clinical trials. In addition, brain organoid and assembloid cultures accurately recapitulate processes of neurodevelopment, disruptions to which can be measured down to the single cell level. Combined, these data point to highly connected networks of shared organismal functions that primarily affect specific neuronal cell subtypes. While current gene therapy development focuses on a minority of NDD cases with the most pronounced phenotypes, these shared mechanisms suggest that treatments developed for these severe cases may also benefit related but less severe cases in the future. Though few clinical trials are currently in progress for NDDs using gene editing technologies, there is promising preclinical data from *in vitro* and animal models for many monogenic and syndromic forms. These extraordinary advances in our understanding of the genetic underpinnings of NDDs and the development and trial of gene editing therapeutics establish a scalable framework that shows tremendous promise in accelerating development of future precision therapies in neurodevelopmental disorders.

## Figures and Tables

**Table 1:** Summary of NDD characterization studies discussed in this review.

Reference	Sample Type	Technique	Result

[98]	Postmortem ASD brain	Bulk RNA	First definition of molecular pathology in ASD or any psychiatric disorder. Identified ASD-associated neuronal immune-glial modules.
[65]	Neurotypical fetal brain	Bulk RNA	ASD risk genes are enriched in neocortical development, most prominently glutamatergic layer 2/3 neurons.
[106]	Neurotypical brain	Bulk RNA	Seeded networks from nine ASD genes identify dysregulation of deep layer projection neurons.
[108]	Postmortem ASD brain	miRNA	Identified miRNAs and co-regulated modules perturbed in ASD
[91]	Postmortem ASD brain	H3K27ac ChIP-seq	greater than 2/3 of syndromic and idiopathic ASD cases share a common acetylome signature
[76]	Postmortem ASD brain	Bulk RNA, miRNA, DNAm, H3K27ac	Two ASD subtypes defined by transcriptomic and epigenetic signatures
[71]	iPSC-derived ASD neurons	Immunoprecipitation	ASD perturbed genes are enriched in layer 2/3 cortical neurons
[36]	iPSC-derived SCZ neurons	Immunoprecipitation	SCZ-perturbed genes are downregulated in layer 5/6 cortical neurons
[23]	Postmortem ASD, BD, SCZ, and neurotypical brain	Bulk RNA, Microarray	Disease-specific and shared expression modules; Shared modules include increased inflammation and excitatory neuron signaling

## Data Availability

No data was used for the research described in the article.

## Abbreviations:

ASD: Autism Spectrum Disorders
SCZ: Schizophrenia
BD: Bipolar Disorder
NDD: Neurodevelopmental Disorders
GWAS: Genome Wide Association Study
WGCNA: Weighted Gene Co-Expression Network Analysis
PPI: Protein-Protein Interaction
EEG: Electroencephalogram
MEG: Magnetoencephalography
iPSC: Induced Pluripotent Stem Cell
ASO: Antisense Oligonucleotide
AAV: Adeno-associated Virus
